# Uridine-Cytidine Kinase 2 (UCK2)/Uridine-Cytidine Kinase Like 1 (UCKL1) complex exacerbates the differentiation of myocardial fibroblasts via TRIM21/Smurf2/Smad3 pathway after myocardial infarction

**DOI:** 10.1186/s43556-025-00397-x

**Published:** 2025-12-29

**Authors:** Xiao Zhou, Yu Zhang, Hao Wang, Zhen Qi, Ziyi Gu, Jun Cui, Zhenlei Hu, Yongyi Wang

**Affiliations:** 1 Department of Cardiovascular Surgery, Renji Hospital, School of Medicine, Shanghai Jiao Tong University, Shanghai, China; 2 Department of Anesthesiology, Renji Hospital, School of Medicine, Shanghai Jiao Tong University, Shanghai, China; 3 Department of General Internal Medicine, West China Second University Hospital, Sichuan University, Chengdu, China; 4 Key Laboratory of Birth Defects and Related Diseases of Women and Children (Sichuan University), Ministry of Education, Chengdu, China; 5 Department of Geriatrics, Xijing Hospital, The Fourth Military Medical University, Xi’an, China; 6 Department of Cardiovascular Surgery, Shanghai Ninth People’s Hospital, School of Medicine, Shanghai Jiao Tong University, Shanghai, China

**Keywords:** Myocardial infarction, Myocardial fibrosis, Cardiac fibroblasts, UCK2, UCKL1

## Abstract

Cardiac fibrosis following myocardial infarction (MI) is a pivotal driver of ventricular dysfunction and heart failure, yet the molecular checkpoints orchestrating the persistent activation of cardiac fibroblasts remain incompletely defined. Here, we uncover a non-canonical, metabolism-independent function of Uridine-Cytidine Kinase 2 (UCK2) and Uridine-Cytidine Kinase Like-1 (UCKL1) as synergistic regulators of pathological remodeling. We demonstrate that both proteins are robustly upregulated in the border zone of ischemic murine hearts and transforming growth factor-β (TGF-β)-activated human cardiac fibroblasts (HCFs). Mechanistically, UCK2 and UCKL1 physically assemble into an obligate functional complex that acts as a molecular scaffold rather than a metabolic enzyme. This complex recruits the E3 ubiquitin ligase Tripartite Motif Containing 21 (TRIM21) to orchestrate the specific ubiquitination and degradation of the negative regulator SMAD Specific E3 Ubiquitin Protein Ligase 2 (Smurf2), thereby sustaining SMAD Family Member 3 (Smad3) phosphorylation and amplifying fibrogenic TGF-β signaling. Disruption of this axis via combined genetic silencing exerts a synergistic protective effect by abrogating myofibroblast differentiation and extracellular matrix production. Furthermore, therapeutic intervention using adeno-associated virus (AAV)-mediated knockdown of UCK2/UCKL1 significantly attenuates adverse ventricular remodeling, limits scar expansion, and preserves cardiac function in a murine MI model. Collectively, these findings identify the UCK2/UCKL1-TRIM21-Smurf2-Smad3 axis as a novel, druggable signalosome linking kinase “moonlighting” functions to transcriptional reprogramming, offering a transformative therapeutic strategy to arrest the progression of post-ischemic heart failure.

**Supplementary Information:**

The online version contains supplementary material available at 10.1186/s43556-025-00397-x.

## Introduction

Cardiac fibrosis is a pervasive pathological hallmark of diverse cardiovascular diseases, most notably myocardial infarction (MI), and serves as a primary precipitant of heart failure [1, 2]. Following coronary occlusion, the resultant ischemic insult triggers extensive cardiomyocyte death within the left ventricular myocardium. This acute loss of contractile units necessitates a reparative response, yet inevitably initiates maladaptive remodeling and scar formation that compromise global cardiac contractility [3, 4]. Ultimately, the irreversible loss of functional parenchyma and the stiffening of the ventricular wall precipitate a decline in global cardiac performance, underscoring the urgent need for therapeutic strategies that can modulate this remodeling process.

The progression of this scarring process-termed cardiac fibrosis-is defined by the aberrant accumulation of extracellular matrix (ECM) proteins within the interstitial microenvironment. This excessive ECM deposition is principally orchestrated by activated cardiac fibroblasts. In the wake of ischemic injury, stressed or dying cardiomyocytes release a complex milieu of paracrine signals, including pro-inflammatory cytokines, chemokines, and growth factors [5, 6]. Among these, TGF-β acts as a master regulator, driving the phenotypic conversion of quiescent fibroblasts into myofibroblasts. These differentiated myofibroblasts exhibit a hyper-proliferative and migratory phenotype, expanding from the infarct core into the border zone, thereby propagating fibrosis throughout the myocardium [7, 8]. Ultimately, this unrestrained fibrotic expansion disrupts myocardial architecture and precipitates profound cardiac dysfunction. Consequently, elucidating the molecular underpinnings of fibroblast activation is imperative for developing targeted therapeutic strategies to arrest cardiac fibrosis and halt the progression to heart failure.

Uridine-cytidine kinases (UCKs) represent a pivotal class of rate-limiting enzymes that catalyze the phosphorylation of uridine and cytidine into their respective monophosphates (UMP and CMP) [9]. This pyrimidine salvage pathway is indispensable for sustaining the nucleotide pools required for efficient RNA and DNA synthesis during cellular proliferation [10]. The mammalian UCK family comprises three distinct members: UCK1, UCK2, and UCKL1. Among them, UCK1 and UCK2 exhibit significant structural conservation, sharing approximately 70% sequence identity [11, 12]. Beyond their canonical metabolic roles, emerging evidence has implicated the UCK family as potent drivers of oncogenesis. For instance, UCK1 expression correlates with clinical prognosis and therapeutic sensitivity to azacytidine in myelodysplastic syndromes [13]. Similarly, UCK2 has been identified as a key accelerator of hepatocellular carcinoma [14], cholangiocarcinoma progression [15] and melanoma metastasis [16], while UCKL1 has been shown to fuel the malignancy of colorectal [17]. Despite these extensive insights into their non-metabolic functions as tumor promoters, the potential involvement of the UCK family in pathological tissue remodeling, specifically cardiac fibrosis, remains completely unexplored.

In this study, we unveil a previously unappreciated, non-canonical function of the pyrimidine salvage enzymes UCK2 and UCKL1, identifying them as critical checkpoints in the pathogenesis of post-infarction cardiac fibrosis. Departing from their classical metabolic roles, we demonstrate that UCK2 and UCKL1 physically assemble into a functional heteromeric complex that acts as a molecular scaffold to potentiate TGF-β signaling. Mechanistically, this complex orchestrates a precise rewiring of the ubiquitin–proteasome system by engaging the TRIM21/Smurf2/Smad3 axis, thereby sustaining the activation of pro-fibrotic cascades. Collectively, our findings define the molecular prerequisites of UCK2/UCKL1-driven fibrosis and establish this signaling node as a promising therapeutic target to halt adverse ventricular remodeling in the post-infarcted heart.

## Results

### UCK2 and UCKL1 are upregulated in cardiac fibroblasts after TGF-β stimulation and myocardial infarction

**Fig. 1:**
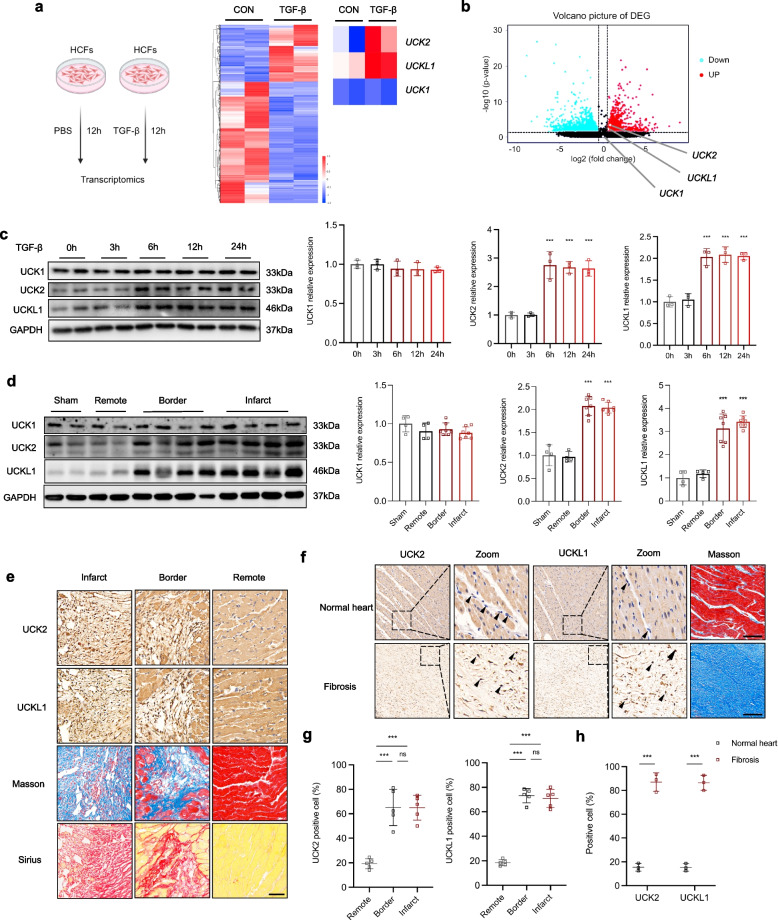
UCK2 and UCKL1 are upregulated in cardiac fibroblasts after TGF-β stimulation and myocardial infarction. **a** Scheme of Transcriptomics and Heatmap showed the key differential genes and UCK family genes after 12 h TGF-β treatment in human cardiac fibroblasts. **b** The red dots represent genes with increased expression after TGF-β treatment in human cardiac fibroblasts, whereas the green dots represent genes with decreased expression. **c** Representative western blotting results of UCK1, UCK2, and UCKL1 in human cardiac fibroblasts after TGF-β treatment and the quantification of immunoblots (n = 3 in each group). **d** Representative western blotting results of UCK1, UCK2, and UCKL1 in myocardial infarction mouse heart tissues and the quantification of immunoblots(n = 4 in each group). **e**, **g** Representative images and quantification of positive cells of mouse heart tissues after myocardial infarction with UCK2, UCKL1 IHC, and Masson, Sirius red staining. Scale bar: 50 μm. **f**, **h** Representative images and quantification of positive cells of human heart tissues after myocardial infarction with UCK2, UCKL1 IHC staining. Scale bar: 100 μm. ***: *p* < 0.0001

To systematically dissect the molecular hierarchy governing cardiac fibroblast activation and differentiation, we performed unbiased transcriptomic profiling on HCFs following TGF-β stimulation. RNA-sequencing analysis revealed a profound transcriptional reprogramming, identifying a distinct gene signature associated with the Uridine-Cytidine Kinase (UCK) family. Strikingly, among family members, UCK2 and UCKL1 were robustly upregulated, whereas UCK1 expression remained largely distinct and unaltered (Fig. 1a, b). To corroborate these transcriptomic findings at the translational level, we assessed protein abundance in both TGF-β-challenged HCFs and ischemic heart tissues following myocardial infarction (MI). Immunoblot analysis confirmed a significant induction of UCK2 and UCKL1 proteins-but not UCK1-in activated fibroblasts, as well as in the fibrotic border and infarct zones of MI hearts (Fig. 1c, d). Spatially, immunohistochemical (IHC) staining further localized the enrichment of UCK2 and UCKL1 specifically to the fibrotic niches of the infarcted myocardium (Fig. 1e, g). Importantly, establishing clinical relevance, we observed a conserved accumulation of UCK2 and UCKL1 in human cardiac fibrosis specimens (Fig. 1f, h). Collectively, these multi-dimensional data implicate UCK2 and UCKL1 as prominent molecular signatures intimately associated with the pathogenesis of cardiac fibrosis.

### UCK2 binds with UCKL1 in cardiac fibroblasts and myocardium

**Fig. 2:**
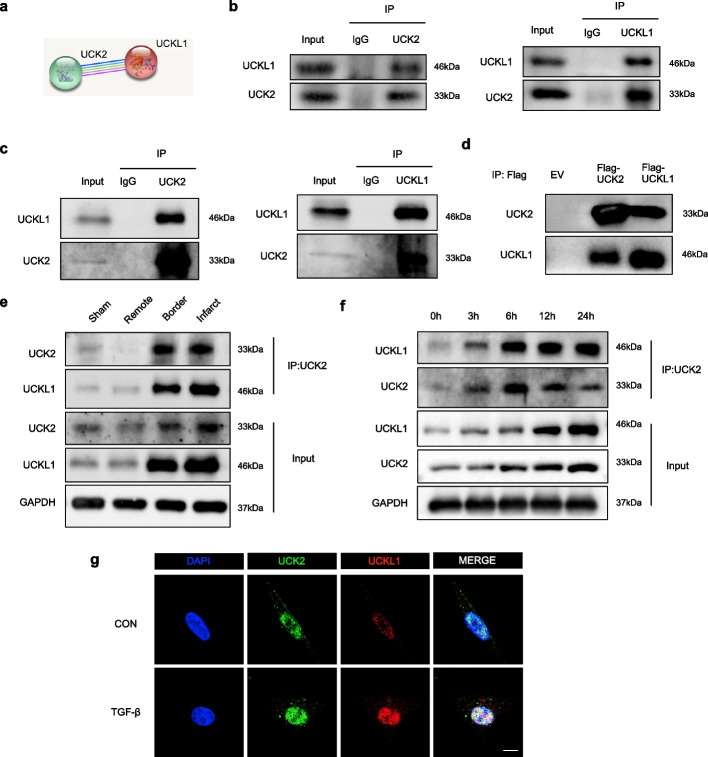
UCK2 binds with UCKL1 in cardiac fibroblasts and myocardium. **a** The interaction of UCK2 and UCKL1 protein in STING database. **b** Coimmunoprecipitation of UCK2 and UCKL1 in human cardiac fibroblasts. **c** Coimmunoprecipitation of UCK2 and UCKL1 in mouse heart tissue. **d** Coimmunoprecipitation of UCK2 and UCKL1 in 293 T cells transfected with Flag-UCK2 and Flag-UCKL1 plasmids. **e** Coimmunoprecipitation of UCK2 and UCKL1 in mouse heart tissue after myocardial infarction. **f** Coimmunoprecipitation of UCK2 and UCKL1 in human cardiac fibroblasts after TGF-β treatment. **g** Colocalization of UCK2 (green) and UCKL1 (red) in human cardiac fibroblasts. Scale bar: 10 μm

To elucidate the molecular interplay between UCK2 and UCKL1, we first performed an in silico analysis using the STRING database, which predicted a high-confidence physical interaction between these two kinase paralogs (Fig. 2a). To experimentally validate this putative interaction, we conducted co-immunoprecipitation (Co-IP) assays, confirming that endogenous UCK2 physically associates with UCKL1 in both HCFs and myocardial tissues (Fig. 2b, c). This direct binding was further corroborated in an exogenous system, where epitope-tagged UCK2 and UCKL1 were shown to co-precipitate in HEK293T cells (Fig. 2d). Intriguingly, quantitative analysis revealed that this protein–protein interaction is dynamic and pathology-dependent; the formation of the UCK2-UCKL1 complex was significantly potentiated in TGF-β-activated HCFs as well as in the infarct and border zones of ischemic hearts (Fig. 2e, f). Complementing these biochemical findings, immunofluorescence (IF) assays demonstrated a distinct subcellular distribution, showing that UCK2 and UCKL1 predominantly colocalize within the nucleus (Fig. 2g). Collectively, these data provide robust evidence that UCK2 and UCKL1 form a stable, stress-responsive nuclear complex in cardiac fibroblasts.

### UCK2 and UCKL1 regulate the differentiation of cardiac fibroblasts

**Fig. 3:**
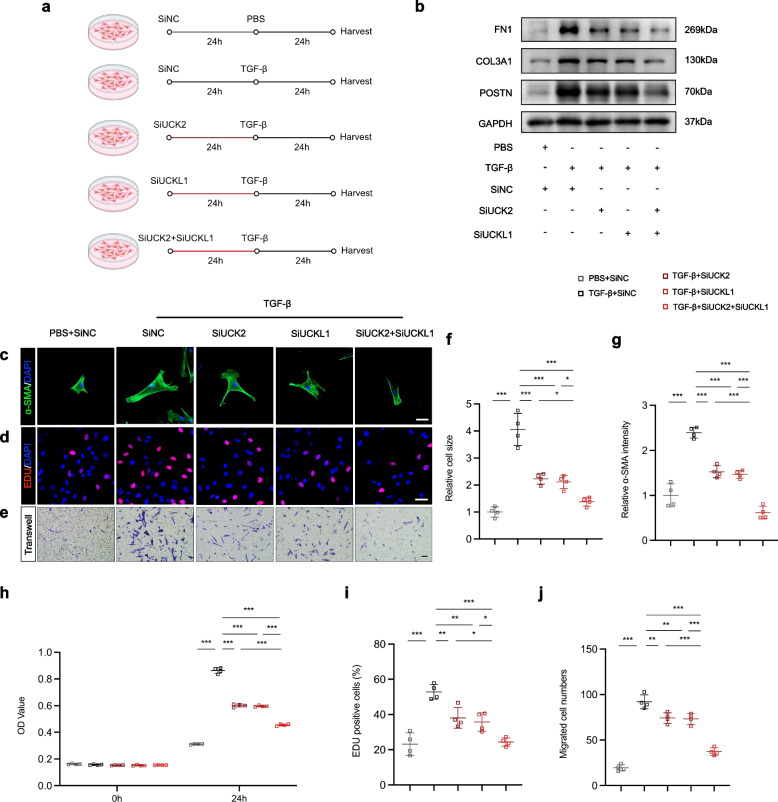
UCK2 and UCKL1 regulate the differentiation of cardiac fibroblasts. **a** Scheme of UCK2 and UCKL1 intervention in HCFs. **b** Representative western blotting results of FN1, COL3A1, and POSTN in human cardiac fibroblasts. **c** α-SMA (green) and DAPI (blue) staining in human cardiac fibroblasts. Scale bar: 50 μm. **d** EDU staining in human cardiac fibroblasts. Scale bar: 50 μm. **e** Transwell assay in human cardiac fibroblasts. Scale bar: 100 μm. **f** The quantification of cell size of (**c**) (n = 4 in each group). **g** The quantification of α-SMA intensity of (**c**) (n = 4 in each group). **h** The OD value of human cardiac fibroblast in CCK-8 assay (n = 4 in each group). **i** The quantification of EDU.^+^ cells of (**d**) (n = 4 in each group). **j** The quantification of migrated cells of (**e**) (n = 4 in each group). *: *p* < 0.05, **: *p* < 0.001, ***: *p* < 0.0001

To delineate the functional necessity of UCK2 and UCKL1 in driving cardiac fibroblast activation and differentiation, we employed a loss-of-function approach using targeted small interfering RNAs (siRNAs) in HCFs (Fig. 3a, Fig. S1a, b). Silencing of either UCK2 or UCKL1 significantly attenuated the TGF-β-induced profibrotic program, as evidenced by the reduced expression of canonical myofibroblast markers, including POSTN, COL3A1, and FN1 (Fig. 3b). Morphologically, immunofluorescence assays revealed that UCK2 or UCKL1 deficiency abrogated TGF-β-driven cellular hypertrophy and stress fiber formation, indicated by decreased cell surface area and α-SMA intensity (Fig. 3c, f, g). Furthermore, functional assays (CCK-8, EdU, and Transwell) consistently demonstrated that depletion of these kinases compromised the proliferative capacity and migratory potential of activated HCFs (Fig. 3d, e, h-j). Most notably, concomitant silencing of both UCK2 and UCKL1 exerted a pronounced additive suppressive effect, leading to a more profound inhibition of marker gene expression and cellular functions compared to single knockdowns (Fig. 3b-j). To rule out off-target interference, we employed alternative siRNA sequences (siUCK2-2 and siUCKL1-2) and observed a comparable reduction in fibrogenic markers (Fig. S2a). Furthermore, the specificity of these effects was confirmed by rescue assays, where the ectopic expression of UCK2 and UCKL1 effectively reversed the downregulation of fibrogenic genes induced by their respective siRNAs (Fig. S2b). Collectively, these results establish UCK2 and UCKL1 as critical, potentially cooperative drivers of TGF-β-mediated HCF differentiation.

### UCK2 and UCKL1 mediate the differentiation of cardiac fibroblasts as a protein complex

**Fig. 4:**
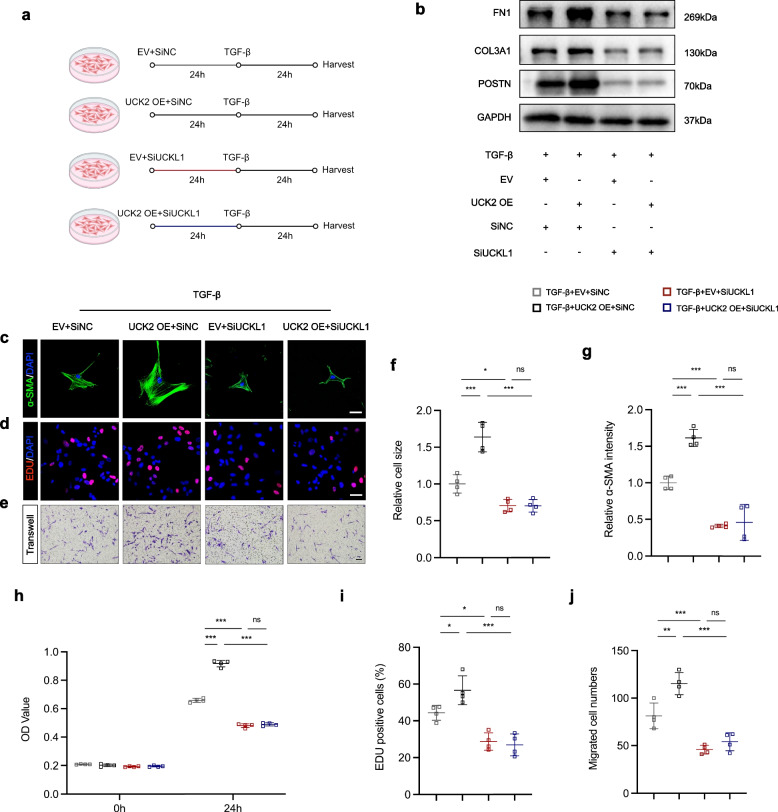
UCK2 and UCKL1 mediate the differentiation of cardiac fibroblasts as a protein complex. **a** Scheme of UCK2 and UCKL1 intervention in HCFs. **b** Representative western blotting results of FN1, COL3A1, and POSTN in human cardiac fibroblasts. **c** α-SMA (green) and DAPI (blue) staining in human cardiac fibroblasts. Scale bar: 50 μm. **d** EDU staining in human cardiac fibroblasts. Scale bar: 50 μm. **e** Transwell assay in human cardiac fibroblasts. Scale bar: 100 μm. **f** The quantification of cell size of (**c**) (n = 4 in each group). **g** The quantification of α-SMA intensity of (**c**) (n = 4 in each group). **h** The OD value of human cardiac fibroblast in CCK-8 assay (n = 4 in each group). **i** The quantification of EDU.^+^ cells of (**d**) (n = 4 in each group). **j** The quantification of migrated cells of (**e**) (n = 4 in each group). *: *p* < 0.05, **: *p* < 0.001, ***: *p* < 0.0001

To investigate the hypothesis that UCK2 acts synergistically with UCKL1 as a functional complex, we manipulated their expression levels in HCFs specifically by transfecting UCK2-overexpression plasmids (pcDNA3.1) and/or silencing UCKL1 via siRNA (Fig. 4a, Fig. S1a). Characterization of the fibrotic phenotype revealed that UCK2 overexpression significantly upregulated the transcription of myofibroblast markers, including POSTN, COL3A1, and FN1 (Fig. 4b). Consistently, immunofluorescence analysis demonstrated that UCK2-overexpressing cells exhibited distinct myofibroblast features, characterized by increased cell surface area and intensified α-SMA stress fibers (Fig. 4c, f, g). Furthermore, functional assays-including CCK-8, EdU incorporation, and Transwell migration-confirmed that UCK2 overexpression markedly potentiated the proliferative and migratory capacities of TGF-β-induced HCFs (Fig. 4d, e, h-j). Conversely, UCKL1 knockdown suppressed these fibrogenic responses. Crucially, we examined the dependency of UCK2 on UCKL1. We found that UCKL1 silencing effectively abrogated the pro-fibrotic effects driven by UCK2 overexpression; HCFs with simultaneous UCK2 upregulation and UCKL1 knockdown displayed significantly reduced differentiation and functional activation compared to cells with UCK2 overexpression alone (Fig. 4b-j). Collectively, these findings indicate that UCKL1 is indispensable for UCK2-mediated signaling, suggesting that they function as an obligate protein complex to drive pro-fibrotic responses.

### UCK2 and UCKL1 mediate the differentiation of cardiac fibroblasts via TRIM21

**Fig. 5:**
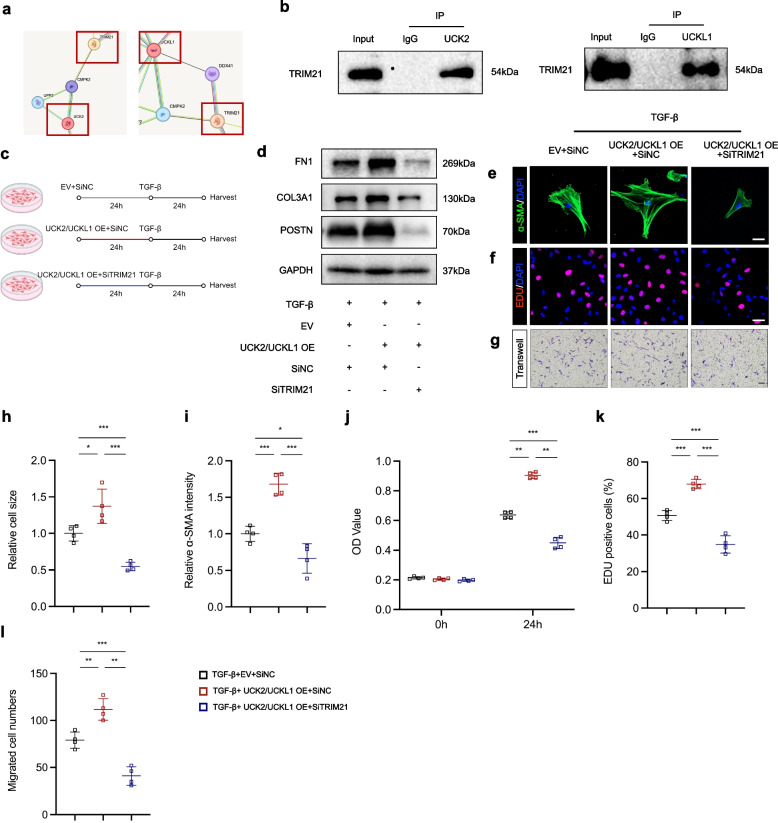
UCK2 and UCKL1 mediate the differentiation of cardiac fibroblasts via TRIM21. **a** The interaction of UCK2, UCKL1, and TRIM21 in the STRING database. **b** Co-IP of UCK2, UCKL1, and TRIM21 in human cardiac fibroblasts. **c** Scheme of UCK2, UCKL1 and TRIM21 intervention in HCFs. **d** Representative western blotting results of FN1, COL3A1, and POSTN in human cardiac fibroblasts. **e** α-SMA (green) and DAPI (blue) staining in human cardiac fibroblasts. Scale bar: 50 μm. **f** EDU staining in human cardiac fibroblasts. Scale bar: 50 μm. **g** Transwell assay in human cardiac fibroblasts. Scale bar: 100 μm. **h** The quantification of cell size of (e) (n = 4 in each group). **i** The quantification of α-SMA intensity of (**e**) (n = 4 in each group). **j** The OD value of human cardiac fibroblast in CCK-8 assay (n = 4 in each group). **k** The quantification of EDU.^+^ cells of (**f**) (n = 4 in each group). **l** The quantification of migrated cells of (**g**) (n = 4 in each group). *: *p* < 0.05, **: *p* < 0.001, ***: *p* < 0.0001

To elucidate the molecular mechanism driving UCK2/UCKL1-mediated HCFs activation, we screened for interacting partners using the STRING database and identified TRIM21 as a putative binding partner (Fig. 5a). We validated this physical association via co-IP assays, confirming that both UCK2 and UCKL1 form a complex with TRIM21 (Fig. 5b). To determine whether TRIM21 is functionally required for UCK2/UCKL1 signaling, we performed rescue experiments by co-transfecting UCK2/UCKL1 overexpression plasmids while silencing TRIM21 in HCFs (Fig. 5c, Fig. S1c). Phenotypic characterization revealed that UCK2 and UCKL1 overexpression significantly aggravated myofibroblast differentiation, evidenced by the upregulation of POSTN, COL3A1, and FN1 (Fig. 5d), as well as cellular hypertrophy and enhanced α-SMA organization (Fig. 5e, h, i). Furthermore, this overexpression robustly promoted TGF-β-induced proliferation and migration (Fig. 5g, j–l). Importantly, these pro-fibrotic effects were effectively abrogated by TRIM21 knockdown. HCFs with simultaneous UCK2/UCKL1 overexpression and TRIM21 silencing exhibited reduced fibrotic marker expression, cell size, and proliferative/migratory capacities compared to overexpression alone (Fig. 5d–l). Collectively, these data demonstrate that UCK2 and UCKL1 exert their pro-fibrotic effects in a TRIM21-dependent manner.

### UCK2/UCKL1/TRIM21 complex regulates ubiquitin and expression of Smurf2 and p-Smad3

**Fig. 6:**
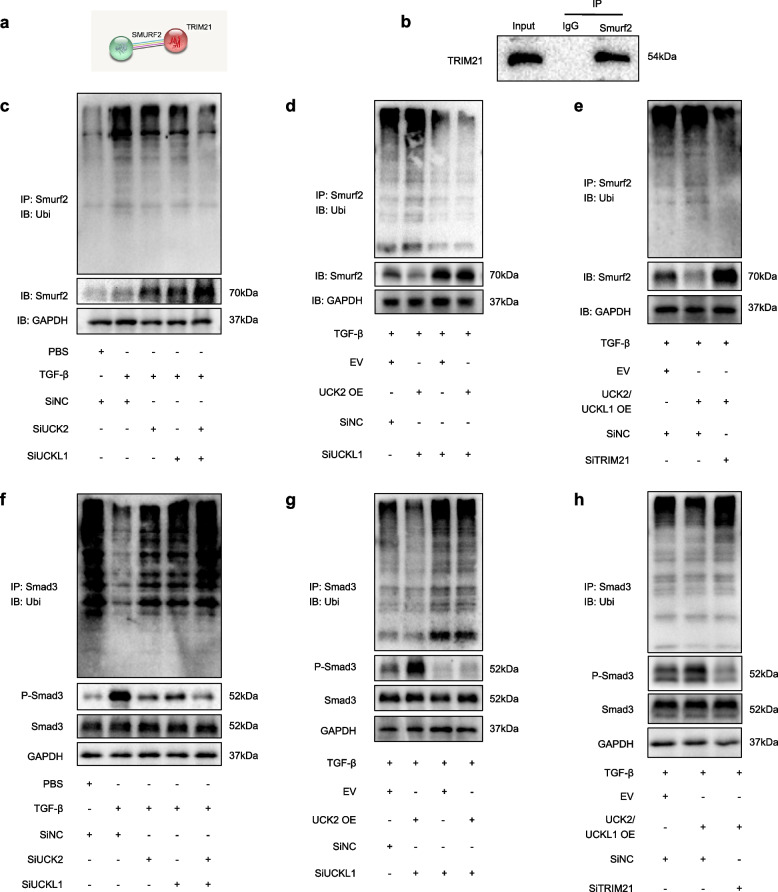
UCK2/UCKL1/TRIM21 complex regulates ubiquitin of Smad3 and Smurf2. **a** The interaction of TRIM21 and Smurf2 in the STING database. **b** co-IP of TRIM21 and Smurf2 in human cardiac fibroblasts. **c**, **d**, **e** Lysates of human cardiac fibroblast were subjected to Co-Ip with anti-Smurf2, which was followed by immunoblot with anti-Ubi. **f**, **g**, **h** Lysates of human cardiac fibroblast were subjected to Co-Ip with anti-Smad3, which was followed by immunoblot with anti-Ubi

Given the role of TRIM21 as an E3 ubiquitin ligase, we hypothesized that the UCK2/UCKL1/TRIM21 complex modulates the stability of key TGF-β pathway components Following in silico prediction (STRING) and experimental validation via co-IP assays (Fig. 6a, b), we identified Smurf2 as a direct physical interactor of TRIM21. To dissect the regulatory dynamics, we performed parallel analyses of ubiquitination profiles (K48) and protein expression levels. We observed a concordant regulation where the knockdown of UCK2 or UCKL1 significantly attenuated the ubiquitination and increased the protein abundance of Smurf2 (Fig. 6c). Conversely, UCK2 overexpression potentiated Smurf2 ubiquitination and attenuated protein accumulation (Fig. 6d); crucially, however, these effects were strictly dependent on the integrity of the complex, as silencing either UCKL1 effectively abrogated the UCK2-driven upregulation of Smurf2 ubiquitination and downregulation of Smurf2 protein levels (Fig. 6d). Furthermore, Co-overexpression of UCK2 and UCKL1 enhanced Smurf2 ubiquitination and suppressed its protein accumulation, effects that were abolished upon TRIM21 knockdown (Fig. 6e). Intriguingly, the biochemical status of Smad3 displayed a reciprocal pattern to that of Smurf2 across all conditions: interventions that promoted Smurf2 ubiquitination and stability were associated with a marked reduction in Smad3 ubiquitination and a consequent suppression of p-Smad3 expression (Fig. 6f–h). To uncouple the canonical metabolic functions of UCK2 and UCKL1 from their potential scaffolding roles, we utilized kinase-dead mutants. Strikingly, the loss of kinase activity did not abolish their pro-fibrotic potential, as indicated by the sustained upregulation of fibrogenic markers and HCFs proliferation (Fig. S3). Furthermore, to unequivocally establish the consequence of TRIM21-mediated ubiquitination, we performed CHX chase assays. We found that TRIM21 expression led to a rapid decline in protein stability, significantly decreasing the protein half-life, thereby confirming a mechanism of ubiquitination-mediated degradation (Fig. S4a, b). To verify whether the UCK2/UCKL1 complex accelerates fibroblast activation specifically through TRIM21-mediated ubiquitination, we employed a rescue assay with a TRIM21 loss-of-function mutant (TRIM21-C12A) that lacks ubiquitin ligase activity. Ectopic expression of wild-type TRIM21 (TRIM21-WT) rescued the downregulation of fibrotic genes induced by UCK2/UCKL1 knockdown, whereas the TRIM21-C12A mutant failed to do so (Fig. S4c-e). To validate these findings at the transcriptome level, we performed RNA-seq analysis in UCK2/UCKL1-knockdown cells upon TGF-β stimulation. Consistent with our biochemical data, pathway enrichment analysis highlighted extracellular matrix signaling, TGF-β signaling and Smad protein transduction as the top affected pathways, reinforcing the conclusion that UCK2/UCKL1 governs Smad3-dependent fibrotic responses (Fig. S5). Collectively, these findings indicate that the UCK2/UCKL1 complex recruits TRIM21 to orchestrate the ubiquitination and stabilization of Smurf2, thereby modulating the downstream p-Smad3 signaling axis.

### AAV-mediated UCK2 and UCKL1 knockdown ameliorate cardiac dysfunction and cardiac fibrosis

**Fig. 7:**
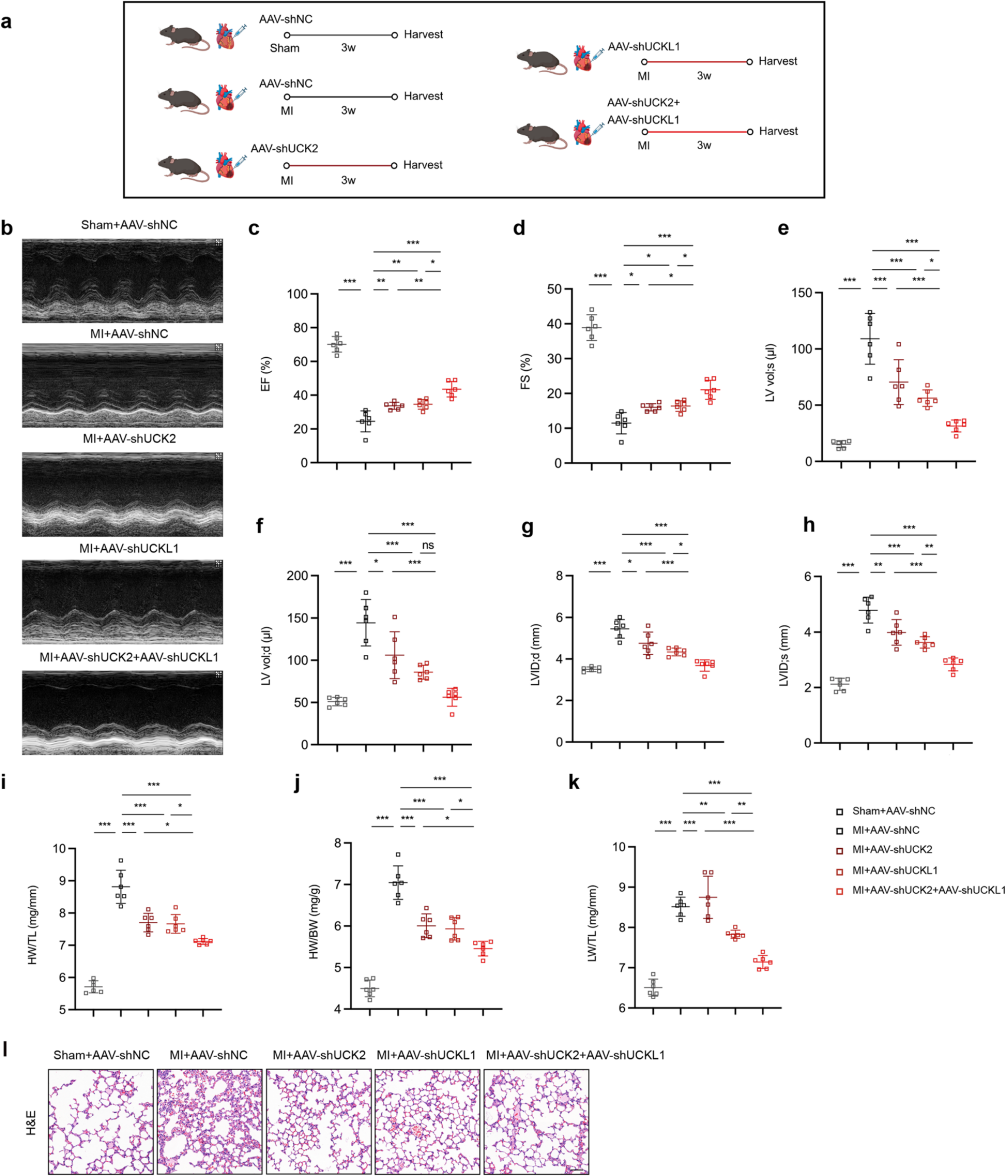
AAV-mediated UCK2 and UCKL1 knockdown ameliorate cardiac dysfunction and cardiac hypertrophy following myocardial infarction. **a** Experimental plan. **b** Representative echocardiographic image from each group in mice. **c**-**h** The quantification of Ejection Fraction (EF), Fractional Shortening (FS), Left Ventricular volume at end-systole (LV vol;s), Left Ventricular volume at end-diastole (LV vol;d), Left Ventricular Internal dimension at end-diastole (LVID;d) and Left Ventricular Internal dimension at end-systole (LVID;s) (n = 6 in each group). **i**-**k** The ratio of heart weight to tibial length, heart weight to body weight, and lung weight to tibial length (n = 6 in each group). **l** Representive images of lung tissue.*: *p* < 0.05, **: *p* < 0.001, ***: *p* < 0.0001

To evaluate the therapeutic potential of targeting the UCK2/UCKL1 axis in vivo, we engineered adeno-associated virus (AAV2/9) vectors driven by the Postn promoter (AAV-shUCK2 and AAV-shUCKL1) to specifically silence these targets in myofibroblasts within the infarcted heart (Fig. S6a, b). In a murine model of myocardial infarction (MI), individual knockdown of either UCK2 or UCKL1 significantly ameliorated pathological remodeling, evidenced by preserved cardiac function, attenuated hypertrophy, and reduced pulmonary edema (Fig. 7a–l). Notably, corroborating our in vitro findings, simultaneous ablation of both UCK2 and UCKL1 yielded superior therapeutic efficacy, resulting in maximal preservation of cardiac performance and structural integrity compared to single-gene targeting.

**Fig. 8:**
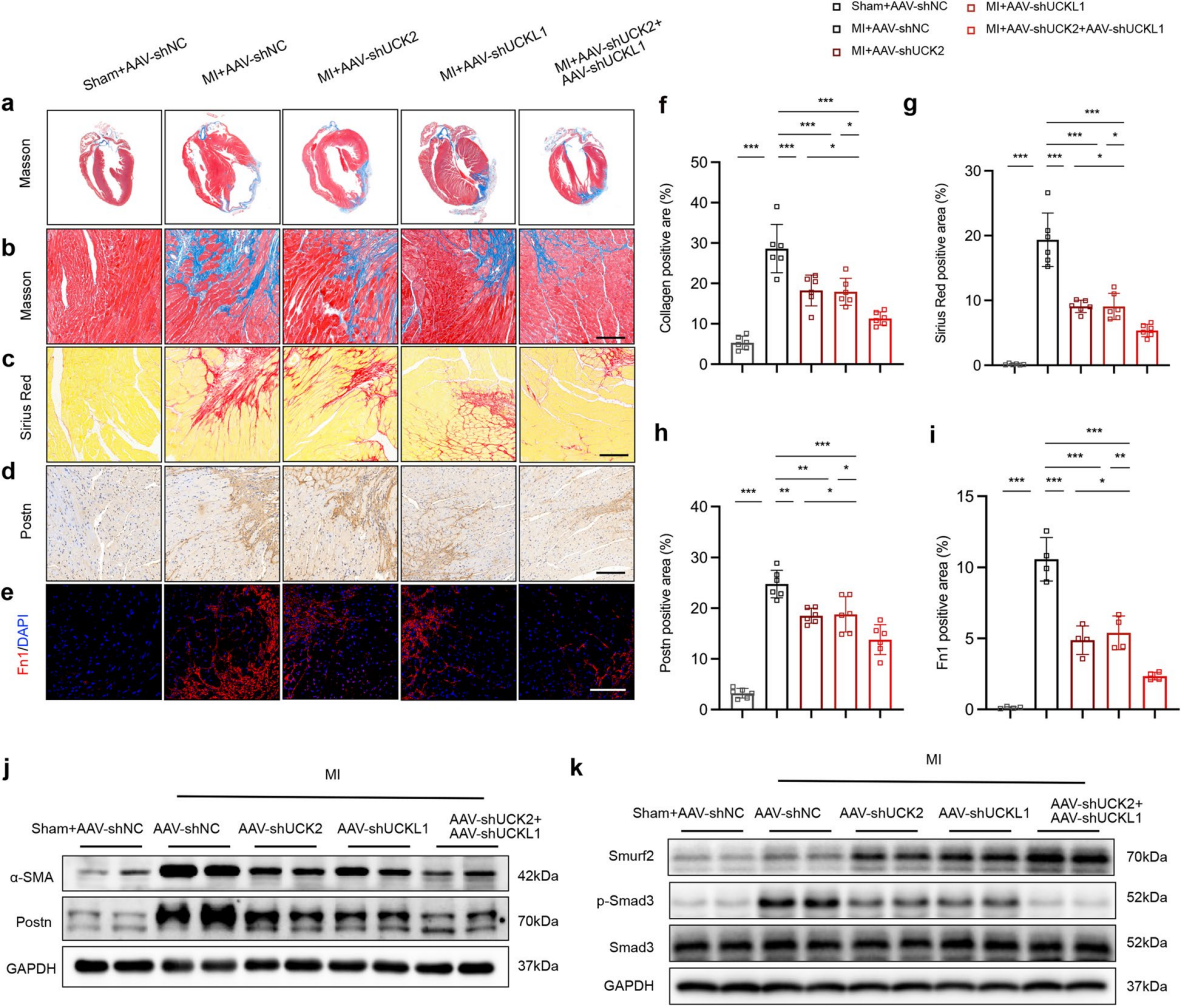
AAV-mediated UCK2 and UCKL1 knockdown attenuate cardiac fibrosis after myocardial infarction. **a**-**b** Representative images of mouse heart tissues after myocardial infarction with Masson staining. Scale bar: 50 μm. **c** Representative images of mouse heart tissues after myocardial infarction with Sirius Red staining. Scale bar: 50 μm. **d** Representative images of mouse heart tissues after myocardial infarction with α-Postn IHC staining. Scale bar: 50 μm. **e** Representative images of mouse heart tissues after myocardial infarction with Fn1 IF staining. Scale bar: 50 μm. **f** The quantification of (**b**) (n = 6 in each group). **g** The quantification of (**c**) (n = 6 in each group). **h** The quantification of (**d**) (n = 6 in each group). **i** The quantification of (**e**) (n = 4 in each group). **j**-**k** Representative western blots of mouse heart tissues after myocardial infarction. *: *p* < 0.05, **: *p* < 0.001, ***: *p* < 0.0001

Histological assessment further confirmed that while AAV-shUCK2 or AAV-shUCKL1 treatment alone limited scar expansion in the infarct border zone, the dual-knockdown strategy led to a significantly more pronounced reduction in cardiac fibrosis, while no alteration of fibrosis was detected in infarct zone (Fig. 8a–c, f, g). Conversely, no significant alteration of fibrosis was detected within the core infarct zone (Fig. S6c). Consistent with this, molecular analysis revealed a robust suppression of myofibroblast activation markers, including α-SMA, Postn, and Fn1. Strikingly, the concurrent silencing of UCK2 and UCKL1 exerted the most potent inhibition on the fibrogenic program (Fig. 8d–e, h–j). To further investigate the regulatory axis in vivo, we assessed the expression levels of Trim21, Smurf2, and p-Smad3. Silencing of UCK2 and UCKL1 led to a significant downregulation of both Smurf2 and p-Smad3, whereas Trim21 expression remained largely unaltered. These results suggest that UCK2 and UCKL1 likely modulate Smurf2 ubiquitination not by affecting TRIM21 expression, but rather through enhancing Trim21’s E3 ubiquitin ligase activity (Fig. 8k, Fig. S6d). Collectively, these findings demonstrate that myofibroblast-specific expression of UCK2 and UCKL1 acts synergistically to drive pathological fibrogenesis and cardiac dysfunction following MI.

## Discussion

Cardiac fibrosis is a hallmark of maladaptive repair post-MI, yet the checkpoints governing fibroblast activation remain incompletely defined. Here, we identify UCK2 and UCKL1 as critical, synergistic drivers of pathological remodeling. Both proteins are markedly upregulated in ischemic myocardium and activated fibroblasts, distinct from the constitutively expressed UCK1. Their fibroblast-specific ablation significantly ameliorates fibrosis and preserves cardiac function in MI models. Mechanistically, we demonstrate that UCK2 and UCKL1 form an obligate functional complex that recruits the E3 ubiquitin ligase TRIM21. Through a hierarchical ubiquitin cascade, this axis targets Smurf2, a key negative regulator of Smad3, for degradation. This action sustains nuclear Smad3 phosphorylation, ultimately amplifying TGF-β signaling. Collectively, our findings establish the UCK2/UCKL1-TRIM21 axis as a potent, druggable regulator of cardiac fibrosis.

We identified a distinct pathological signature characterized by the aberrant upregulation of UCK2 and UCKL1 in ischemic myocardium and activated fibroblasts, distinct from the constitutively expressed isoform UCK1. This specificity positions the UCK2/UCKL1 axis as a dedicated driver of maladaptive remodeling. Therapeutically, myofibroblast-specific ablation of these kinases effectively halted the fibrogenic program and mitigated cardiac dysfunction. Central to our findings is the discovery of a non-redundant, synergistic partnership between UCK2 and UCKL1. The observation that UCKL1 depletion completely abrogates UCK2-mediated fibrosis-and that combined silencing yields superior efficacy-suggests the formation of an obligate functional complex rather than independent parallel signaling. This molecular co-dependency mirrors mechanisms observed in oncology, where the UCK2 and UCKL1 support the metabolic and proliferative demands of tumorigenesis [15, 17, 18]. Crucially, our data using kinase-dead mutants reveals that this pro-fibrotic function is independent of their canonical enzymatic activity in pyrimidine metabolism. Instead, the UCK2/UCKL1 complex acts as a molecular scaffold. By physically interacting with TRIM21, the complex likely potentiates its E3 ligase activity or facilitates precise substrate recruitment. This finding is significant as it uncouples the pathological signaling function from essential metabolic housekeeping, offering a therapeutic window to target the fibrotic driver without disrupting cellular nucleotide homeostasis.

Ubiquitination serves as a pivotal post-translational modification governing protein stability and signal transduction [19, 20], with emerging evidence highlighting its critical role in driving myofibroblast differentiation [21–23]. Here, we identified the E3 ubiquitin ligase TRIM21 (Ro52) as a central effector of the UCK2/UCKL1 axis. While TRIM21 is canonically recognized for its roles in innate immunity, metabolism, and oncogenesis [24–31], recent studies have begun to unveil its pathological contribution to fibrotic diseases [32, 33]. Our study fundamentally advances this understanding by demonstrating a physical and functional convergence between the UCK2/UCKL1 complex and TRIM21. Crucially, TRIM21 proved indispensable for UCK2/UCKL1-mediated signaling, as its depletion effectively negated the pro-fibrotic phenotype driven by kinase overexpression. A striking feature of this interaction was the decoupling of protein abundance from functional output: silencing the UCK2/UCKL1 axis left TRIM21 protein levels unperturbed. This finding argues against a mechanism of stability regulation; instead, it supports a model wherein the UCK2/UCKL1 complex functions as a molecular scaffold. By spatially organizing the signaling node, the complex likely potentiates the E3 ligase activity of TRIM21 or facilitates its precise recruitment to substrates, thereby amplifying the fibrogenic cascade.

To delineate the distal effectors of the UCK2/UCKL1-TRIM21 axis, we employed an integrative approach combining in silico interactomics with rigorous biochemical validation, ultimately establishing Smurf2 as a critical downstream substrate. Canonically, Smurf2 acts as a pivotal ‘molecular brake’ on the TGF-β pathway, maintaining tissue homeostasis by orchestrating the ubiquitin-proteasomal degradation of Smad3 [34–37]. While this protective function of Smurf2 is evolutionarily conserved and well-documented in renal and hepatic fibrosis models [38–40], the upstream molecular hierarchy governing Smurf2 stability-particularly within the distinct microenvironment of the failing heart—has remained elusive. Our study bridges this gap by identifying the UCK2/UCKL1 complex as a novel master regulator of this checkpoint. Distinct from previous reports that focused solely on Smurf2-Smad3 interactions, we unveil a hierarchical ‘E3 ligase cascade’ wherein UCK2 and UCKL1 hijack TRIM21 to target Smurf2 itself for degradation. This mechanism effectively dismantles the intrinsic negative feedback loop: by destabilizing the inhibitor (Smurf2), the UCK2/UCKL1 axis creates a permissive environment for sustained Smad3 activation, thereby locking cardiac fibroblasts into a hyper-active, fibrogenic state.

Our investigation substantiates a mechanistic paradigm wherein the UCK2/UCKL1-TRIM21 axis orchestrates Smurf2 ubiquitination to dictate Smad3 signaling dynamics. A salient and unexpected feature of this regulation emerged from our subcellular fractionation studies, which unveiled a distinct nuclear enrichment of UCK2 and UCKL1 in cardiac fibroblasts. This spatial confinement provides a critical mechanistic insight into our observation that the complex preferentially sustains the levels of phosphorylated Smad3 (p-Smad3), while leaving the total Smad3 pool largely unaffected. Existing literature establishes that Smad3 phosphorylation and its subsequent transcriptional engagement are fundamentally nuclear events; moreover, Smurf2 is known to possess a kinetic preference for targeting the activated, nuclear pool of p-Smad3 for degradation [34, 41, 42]. By integrating our findings with this established canon, we propose a spatially compartmentalized model of signal amplification. In this scenario, the nuclear residency of the UCK2/UCKL1 complex allows it to intercept Smurf2 precisely at the site of Smad3 action. By locally depleting nuclear Smurf2, the complex effectively ‘shields’ the active p-Smad3 from degradation. Thus, rather than regulating the global abundance of Smad3, the UCK2/UCKL1 axis functions as a nuclear rheostat, fine-tuning the delicate equilibrium between phosphorylation and ubiquitination to maximally sustain the transcriptional competency of the fibrogenic program.

Notwithstanding the significance of our findings, several limitations warrant discussion. First, while our study establishes the non-canonical scaffolding function of the UCK2/UCKL1 complex, UCK2 is canonically known for its role in the pyrimidine salvage pathway. Although our data suggest that the fibrotic phenotype is primarily driven by the interaction with TRIM21 rather than metabolic flux, we cannot entirely rule out the possibility that alterations in nucleotide metabolism contribute to the observed therapeutic benefits. Second, our in vivo validation relied primarily on AAV-mediated knockdown in a murine model of MI. Given the inherent physiological and immunological differences between rodents and humans, validation in large-animal models or distinct etiologies of heart failure is necessary to generalize the translational potential. Finally, while AAV vectors served as a robust proof-of-concept tool, the development of small-molecule inhibitors disrupting the UCK2-UCKL1 protein–protein interaction represents a more viable strategy for clinical application.

In conclusion, our study delineates a previously unrecognized mechanism driving post-infarction cardiac fibrosis, governed by the UCK2/UCKL1 signaling axis. We demonstrate that this complex acts as a critical checkpoint for myofibroblast differentiation and pathological remodeling in vivo. Mechanistically, we identify TRIM21 as an essential downstream effector that couples UCK2/UCKL1 activity to the ubiquitin-mediated regulation of the Smurf2/Smad3 axis, thereby amplifying TGF-β signaling. Given that maladaptive fibrosis is a principal determinant of heart failure following myocardial infarction, targeting the UCK2/UCKL1-TRIM21 regulatory node offers a promising therapeutic strategy to arrest fibrogenesis and improve clinical outcomes in ischemic heart disease.

## Method

### Human samples

Human left ventricular (LV) myocardial tissue was obtained from explanted hearts of patients with end-stage ischemic cardiomyopathy undergoing heart transplantation. Fibrotic tissue samples were specifically dissected from the infarct border zones. Non-failing control hearts were procured from male organ donors (cause of death: brain-stem hemorrhage) whose hearts were deemed unsuitable for transplantation for non-cardiac reasons. Detailed clinical demographics and characteristics for the cohort are summarized in Table S1. All procedures were conducted following the principles outlined in the Declaration of Helsinki. Informed consent for heart tissue collection was obtained from all participants and approved by the Medical Ethical Committee of Renji Hospital, Shanghai Jiao Tong University School of Medicine (No. KY2025-001-B). All patient information was de-identified.

### MI model and AAV injection

We used 8–10-week-old male C57BL/6 mice obtained from Youshu Life Technology animal room. All animal experiments were designed and strictly performed by our research team members of Renji Hospital. The animals were housed and maintained at the accredited facility of Youshu Life Technology Co., Ltd. All experimental procedures and protocols were conducted in strict accordance with the Guide for the Care and Use of Laboratory Animals and complied with the ethical standards and regulations of Renji Hospital, School of Medicine, Shanghai Jiao Tong University. The study protocols were reviewed and approved by the Institutional Animal Care and Use Committee (IACUC) of Youshu Life Technology Co., Ltd. (Approval No. YS-m202305003). They were maintained under standard conditions (temperature: 24 ± 2 °C, humidity: 50–60%, 12-h light/dark cycle) with unlimited access to food and water. For the procedures, the mice were anesthetized with pentobarbital at a dosage of 50 mg/kg, administered intraperitoneally. They were then placed on a heated blanket to maintain their body temperature. Respiratory function was supported using a rodent ventilator through tracheal intubation. A precise incision was made between the fourth and fifth intercostal spaces, and the pericardium was carefully opened to expose the left anterior descending coronary artery. Myocardial infarction (MI) was induced by permanently ligating the left anterior descending coronary artery with a 7–0 suture. After this procedure, the thoracic cavity was closed, and both the muscle and skin layers were sutured. Mice in the sham group underwent all the same surgical procedures as those in the MI group, but without ligating the coronary artery. Viral vectors (AAV-POSTN-shNC, AAV-POSTN-shUCK2, and AAV-POSTN-shUCKL1, provided by Hanbio, China) were injected intramyocardially at three points in the peri-infarct zone immediately after the MI surgery. All experiments were randomized, and researchers were blinded to the treatment groups through numerical coding of the mice and samples. Animals that died during surgery or failed to develop myocardial infarction were excluded from the study. Successful myocardial infarction (MI) induction was confirmed intraoperatively by immediate visual blanching of the left ventricular anterior wall following LAD ligation. To ensure a consistent infarct model, echocardiography was performed at 24 h post-surgery, and animals with a Left Ventricular Ejection Fraction (LVEF) > 45% were excluded to maintain a homogeneous cohort with significant ischemic injury. Only animals with similar body weights and normal physiological indices during surgery were analyzed.

### Echocardiography

Mice were anesthetized via inhalation of 1–2% isoflurane in oxygen (flow rate: 1 L/min) and positioned supinely on a heated platform to maintain a physiological body temperature of ~ 37 °C. Hair over the precordium was removed using a chemical depilatory to minimize signal attenuation. Transthoracic echocardiography was performed using a high-resolution Vevo 3100 imaging system equipped with a linear array transducer (FUJIFILM VisualSonics). High-resolution B-mode images were acquired from the parasternal long-axis view. Cardiac function parameters were quantified using the Vevo LAB 5.6.1 software workstation. To ensure data reliability, measurements were averaged from at least three consecutive cardiac cycles per mouse. Animals were monitored until full recovery from anesthesia.

### Immunoblotting and co-immunoprecipitation (co-IP)

Cell and tissue samples were lysed using RIPA lysis buffer (Beyotime, China), which included protease and phosphatase inhibitor cocktails, also from Beyotime. The total protein concentration was measured using a BCA assay kit (Beyotime, China). Proteins were then separated using SDS-PAGE gels, and the gels were transferred to PVDF membranes at a current of 300 mA for 2 h in an ice water bath. The membranes were incubated for 1 h at room temperature with 5% non-fat milk dissolved in TBST buffer (0.1% v/v Tween-20 in TBS buffer) for blocking. After blocking, the membranes were washed three times with TBST for 10 min each. They were then incubated with primary antibodies overnight at 4 °C. Following another round of washing, the membranes were incubated with secondary antibodies conjugated with HRP (Beyotime, China). Finally, images were captured using the Bio-Rad imaging system and analyzed with ImageJ software (NIH).

For co-immunoprecipitation (co-IP), protein lysates were incubated with primary antibodies at 4 °C overnight. The mixtures were then incubated with protein G IgG Sepharose beads (Beyotime, China) at 4 °C for 2 to 4 h. After discarding the supernatants of the protein lysates, the remaining sediments were washed with PBS. The washed sediments were resuspended in a loading buffer, and immunoblotting was performed.

Primary antibodies: anti-UCK1 (12,271–1-AP; Proteintech) (1:100), anti-UCK2 (10,511–1-AP; Proteintech) (1:100), anti-UCKL1 (17,005–1-AP; Proteintech) (1:100), anti-UCKL1 (sc-100636; Santa Cruz) (1:50), anti-TRIM21 (A1957; Abclonal) (1:100), anti-Smurf2 (sc-518164; Santa Cruz) (1:50), anti-Smad3 (sc-101154; Santa Cruz) (1:50), anti-p-Smad3 (sc-517575; Santa Cruz) (1:50), anti-K48-linkage Specific Ubiquitin (A3606; Abclonal) (1:1000), anti-α-SMA (A17910; Abclonal) (1:500), anti-POSTN (NBP1-82,472; Novus) (1:100), anti-FN1 (A12977; Abclonal) (1:100), anti-GAPDH (A19056; Abclonal) (1:10,000). Secondary antibodies: HRP-labeled Goat Anti-Rabbit IgG (H + L) (A0208; Beyotime) (1:5000), HRP-labeled Goat Anti-Mouse IgG (H + L) (A0216; Beyotime) (1:5000).

### Histology staining

Mouse hearts were harvested following transcardial perfusion with saline to remove residual blood, and subsequently fixed in 4% paraformaldehyde for 24 h. The tissues were then dehydrated through a graded ethanol series and embedded in paraffin. Serial Sects. (5 μm thickness) were prepared and subjected to Masson’s trichrome and Sirius Red staining using commercial kits (Solarbio, Beijing, China) according to the manufacturer’s instructions, to visualize myocardial fibrosis and collagen deposition. Whole-slide digital imaging was performed using an Aperio AT2 Slide Scanner (Leica Biosystems, Wetzlar, Germany). Quantitative analysis of the fibrotic area was conducted using ImageJ software (NIH, Bethesda, MD, USA) by an independent pathologist blinded to the experimental group allocation.

### Immunofluorescence and immunohistochemistry

In the immunofluorescence assay, both the cells and heart tissue Sects. (6 μm) were blocked using 5% BSA (Solarbio, China) and then incubated overnight with primary antibodies at 4 °C. The following morning, the cells were incubated at room temperature for 2 h with secondary antibodies conjugated to fluorophores. The nuclei were counterstained with DAPI (Invitrogen, USA), and images were captured using an Olympus confocal microscope (Japan). For the immunohistochemistry procedure, heart tissue Sects. (8 μm) underwent antigen retrieval and were blocked with 5% BSA for 1 h at room temperature. The sections were then incubated overnight with primary antibodies at 4 °C, followed by incubation with peroxidase-conjugated secondary antibodies (Proteintech). For the chromogenic reaction, 3,3-diaminobenzidine was utilized, and the nuclei were stained with hematoxylin. Coverslips were then imaged using a digital microscope (Leica Microsystems, Germany). All images were obtained with a confocal microscope (Olympus), and the image analyses were performed using ImageJ software (NIH).

Primary antibodies: anti-UCK2 (10,511–1-AP; Proteintech) (1:100), anti-UCKL1 (17,005–1-AP; Proteintech) (1:100), anti-UCKL1 (sc-100636; Santa Cruz) (1:50), anti-α-SMA (A17910; Abclonal) (1:500), anti-POSTN (NBP1-82,472; Novus) (1:100), anti-FN1 (A12977; Abclonal) (1:100). Secondary antibodies: Donkey anti-rabbit Alexa Fluor 488 (A21206, Invitrogen) (1:200), Streptavidin Alexa Fluor 647 (S32357; Invitrogen) (1:200).

### Cell culture

Human cardiac fibroblasts (HCF, Lonza, CC-2904) were cultured using FGM™−3 Cardiac Fibroblast Growth Medium-3 BulletKit™ (Lonza, CC-4526) and were used between passages 3 and 5. All primary cell cultures were routinely tested for mycoplasma contamination using BeyoColor™ Mycoplasma Colorimetric LAMP Assay Kit. We confirm that all cells used in this study tested negative for mycoplasma. Following this, the cells were equilibrated for 24 h in low serum medium and then treated with 10 ng/ml recombinant human TGF-β (R&D Systems, catalogue no. 240-B-002) for the specified durations.

### Cell viability assay

Cell viability was quantitatively assessed using the Cell Counting Kit-8 (CCK-8; Dojindo Laboratories, Kumamoto, Japan). Briefly, HCFs were resuspended in complete growth medium and seeded into 96-well plates at an initial density of 1 × 10^4 cells per well. At the designated time points, the CCK-8 reagent was added to the culture medium, and cell viability was determined by measuring the absorbance at 450 nm using a microplate reader.

### EdU staining assay

Cell proliferation was assessed using the EdU incorporation assay (Beyotime, Shanghai, China). Briefly, cells were incubated with 10 μM 5-ethynyl-2’-deoxyuridine (EdU) in complete growth medium for 2 h at 37 °C. Following labeling, cells were fixed with 4% paraformaldehyde for 15 min and permeabilized with 0.5% Triton X-114 (or 0.3% Triton X-100). The EdU incorporation was visualized via the Click-iT reaction using an Apollo 555 dye, strictly adhering to the manufacturer’s instructions. Nuclei were counterstained with DAPI or Hoechst 33,342. Images were acquired using a confocal microscope (Olympus), and the percentage of EdU-positive nuclei relative to total nuclei was quantified using ImageJ software (NIH).

### Transcriptomics study

Transcriptional profiling was conducted via bulk RNA sequencing to identify differentially expressed genes. Total RNA was extracted from HCFs treated with either TGF-β or PBS (serving as the vehicle control). To guarantee the reliability of downstream analyses, the quantity and integrity of the extracted RNA were rigorously validated utilizing an Agilent 2100 Bioanalyzer. Following quality assurance, poly(A)-enriched cDNA libraries were constructed and sequenced on an Illumina HiSeq 2000 platform. The resulting deep-coverage reads provided a solid foundation for subsequent bioinformatic pipelines, including read mapping, quantification, and differential expression analysis.

### Transwell assay

Cell migration was assessed using 24-well Transwell chambers with 8.0-μm pore size polycarbonate membranes (Corning, NY, USA). HCFs were resuspended in serum-free medium and seeded into the upper chamber, while the lower chamber was filled with 600μL of complete medium containing 10% fetal bovine serum (FBS) as a chemoattractant. After 48 h of incubation at 37 °C, non-migrated cells on the upper surface of the membrane were gently removed with cotton swabs. The migrated cells on the lower surface were fixed with 4% paraformaldehyde for 30 min and stained with 0.2% crystal violet solution. Migration was quantified by capturing images of at least five randomly selected fields per well using an inverted light microscope, and cell numbers were analyzed using ImageJ software (NIH).

### Statistical analysis

The data presented in this study are derived from a minimum of three independent experiments and are expressed as the mean ± standard deviation (SD). The specific sample size (n) for each experiment is detailed, where 'n' represents biological replicates rather than technical replicates. In our in vitro experiments, each dataset reflects the average from multiple cultured cells, and we assume that the data conform to a normal distribution based on the central limit theorem. For in vivo experiments, the sample size was no fewer than four. Group comparisons were analyzed using unpaired two-tailed Student’s t-test or one-way ANOVA analysis with post hoc analysis. A difference was considered statistically significant when *p* < 0.05. All data analyses were conducted using GraphPad Prism 8.0 software (San Diego, California).

## Supplementary Information

Supplementary Material 1.

## Data Availability

The datasets generated and/or analyzed during the current study are not publicly available due to ethical restrictions and patient confidentiality agreements, as well as ongoing data analysis. However, the data may be available from the corresponding author upon reasonable request.
