# Supplementary material for: Uridine-Cytidine Kinase 2 (UCK2)/Uridine-Cytidine Kinase Like 1 (UCKL1) complex exacerbates the differentiation of myocardial fibroblasts via TRIM21/Smurf2/Smad3 pathway after myocardial infarction
Source: Mol Biomed. 2025 Dec 29;6:151. doi: 10.1186/s43556-025-00397-x (PMC12745349; doi:10.1186/s43556-025-00397-x)
Supplement: Supplementary file 1 — Supplementary Material 1. [file 43556_2025_397_MOESM1_ESM.docx]

**Supplemental Materials**

**Uridine-Cytidine Kinase 2 (UCK2)/ Uridine-Cytidine Kinase Like 1 (UCKL1) complex exacerbates the differentiation of myocardial fibroblasts via TRIM21/Smurf2/Smad3 pathway after myocardial infarction**

Xiao Zhou^2†^, Yu Zhang^1†^, Hao Wang^3,4†^, Zhen Qi^1^, Ziyi Gu^1^, Jun Cui^1,5^*, Zhenlei Hu^6^*, and Yongyi Wang^1^*

^1^Department of Cardiovascular Surgery, Renji Hospital, School of Medicine Shanghai Jiaotong University, Shanghai, China

^2^Department of Anesthesiology, Renji Hospital, School of Medicine Shanghai Jiaotong University, Shanghai, China

^3^Department of General Internal Medicine, West China Second University Hospital, Sichuan University

^4^Key Laboratory of Birth Defects and Related Diseases of Women and Children (Sichuan University), Ministry of Education

^5^Department of Geriatrics, Xijing Hospital, The Fourth Military Medical University, Xi’an, China

^6^Department of Cardiovascular Surgery, Shanghai Ninth People’s Hospital, School of Medicine, Shanghai Jiao Tong University

†These authors have contributed equally to this work.

*Correspondence:

Yongyi Wang: wangyongyi@renji.com;

Zhenlei Hu: [13564677103@163.com](mailto:13564677103@163.com);

Jun Cui : cuijun1021@yeah.net

Competing interests: The authors declare no competing interests.

**SFig 1: The protein level of UCK2, UCKL1 and TRIM21 after plasmid transfection.**

(a-c) Representative western blotting results of UCK2, UCKL1, and TRIM21 in human cardiac fibroblasts and the quantification of immunoblots.

**SFig 2: The protein level of FN1 and COL3A1 after UCK2 and UCKL1 intervention.**

(a-b) Representative western blotting results of FN1 and COL3A1 in human cardiac fibroblasts.

**SFig 3: UCK2 and UCKL1 mutants similarly accelerate HCFs activation.**

(a, d) Representative western blotting results of FN1 and COL3A1 in human cardiac fibroblasts. (b, e) The OD value of human cardiac fibroblast in CCK-8 assay (n=4 in each group). (c, f) EDU staining in human cardiac fibroblasts and the quantification of EDU^+^ cells (n=3 in each group). Scale bar: 50μm. *: *p*<0.05, **: *p*<0.001, ***: *p*<0.0001, ns: no significance.

**SFig 4: TRIM21 mutant fails to rescue the differentiation UCK2 and UCKL1 knockdown HCFs.**

(a) Representative western blotting results of Smurf2 in human cardiac fibroblasts. (b) Representative western blotting results of Smad3 in human cardiac fibroblasts. (c) Representative western blotting results of FN1 and COL3A1 in human cardiac fibroblasts. (d) The OD value of human cardiac fibroblast in CCK-8 assay (n=4 in each group). (e) EDU staining in human cardiac fibroblasts and the quantification of EDU^+^ cells (n=3 in each group). Scale bar: 50μm. **: *p*<0.001, ***: *p*<0.0001, ns: no significance.

**SFig 5: Transcriptomics analysis of UCK2/UCKL1 knockdown HCFs and control HCFs after 24 hours TGF-β stimulation.**

(a) KEGG pathway. (b) GO pathway. (c) GSEA analysis with GO database.

**SFig 6: Therapeutic targeting of UCK2 and UCKL1 influences cardiac fibrotic responses.**

(a) UCK2 and Postn staining in myocardial fibrosis heart tissue and the quantification of UCK2^+^Postn^+^/Postn^+^ cells (n=3 in each group). (b) UCKL1 and POSTN staining in myocardial fibrosis heart tissue and the quantification of UCKL1^+^POSTN^+^/POSTN^+^ cells (n=3 in each group). (c) FN1 staining in infarct zone of mouse heart tissue and the quantification of FN1 positive area (n=5 in each group) (d) The OD value of human cardiac fibroblast in CCK-8 assay (n=4 in each group). (e) Representative western blotting results of TRIM21 in heart tissues. Scale bar: 100μm. *: *p*<0.05, **: *p*<0.001.

**Table 1: Clinical characteristics and Blood Biochemistry Data**

|  | Control | MI | *P* value |
| --- | --- | --- | --- |
| Number | 3 | 3 | ns |
| Gender (Male) | 3 | 3 | ns |
| Age (Years old) | 59.33±3.51 | 60.67±5.13 | ns |
| Height (cm) | 171.7±4.52 | 170.8±3.38 | ns |
| Body weight (kg) | 62.33±2.85 | 61.13±4.15 | ns |
| BMI (kg/m^2^) | 21.15±0.77 | 20.94±0.60 | ns |
| Systolic blood pressure (mmHg) | 135.7±5.55 | 134.0±6.06 | ns |
| Diastolic blood pressure (mmHg) | 83.53±10.02 | 85.30±9.283 | ns |
| FBG (mmol/L) | 6.40±0.55 | 6.51±0.89 | ns |
| LDL (mmol/L) | 2.13±0.61 | 2.74±0.69 | ns |
| Total cholesterol (mmol/L) | 4.08±0.63 | 4.74±0.87 | ns |
| Triglyceride (mmol/L) | 1.97±0.39 | 1.41±0.21 | ns |
| Days after infarction (d) | 19.67±7.02 | - | - |

ns: no significance; BMI: body mass index; FBG: fasting blood-glucose; LDL: low density lipoprotein;
